# Patterns and Predictors of Influenza Vaccination Uptake Among Healthcare Personnel in Kuwait: A Nationwide Multi-Season Study

**DOI:** 10.3390/vaccines14080712

**Published:** 2026-08-19

**Authors:** Saleh Alsarhan, Fatema Alkhulaifi, Lolwah Alzafiri

**Affiliations:** 1 Department of Health Policy and Management, College of Public Health, Kuwait University, Kuwait City 13060, Kuwait; 2 Department of Epidemiology and Biostatistics, College of Public Health, Kuwait University, Kuwait City 13060, Kuwait; fatemah.alkhulaifi@ku.edu.kw; 3 Kuwait Center for Disease Control and Prevention (KCDC), Ministry of Health, Kuwait, Kuwait City 13001, Kuwait; alzafiri3941@moh.gov.kw

**Keywords:** health personnel, influenza vaccines, vaccination coverage, sickness absence, healthcare workers, Kuwait

## Abstract

**Background/Objectives**: Healthcare personnel (HP) are a priority group for seasonal influenza vaccination due to their increased occupational exposure and potential to transmit influenza in healthcare settings. This study examined patterns and factors associated with in-season influenza vaccination uptake among HP in Kuwait. **Methods**: A nationwide observational study was conducted using Ministry of Health (MOH) administrative records covering the 2022/2023 through 2024/2025 influenza seasons. A person–season dataset was constructed, comprising one observation per HP for each of the three seasons. The primary outcome was in-season influenza vaccination, defined as receipt of an influenza vaccine between September and March of a given study season. Independent variables included age, sex, nationality, governorate of residence, profession, managerial position, previous-year influenza vaccination status, and pre-season sickness absence duration (days). Generalized estimating equations (GEE) logistic regression model with an exchangeable correlation structure was used to estimate factors associated with vaccination uptake. **Results**: The final analytic dataset included 48,886 HP contributing 146,658 person–season observations across three influenza seasons. Overall, in-season vaccination uptake was 8.7%, and repeated vaccination across seasons was uncommon. Older age, male sex, non-Kuwaiti nationality, and being a physician were independently associated with higher odds of in-season influenza vaccination. Previous-year influenza vaccination was the strongest predictor of current-season vaccination uptake (aOR 2.01, 95% CI 1.87–2.16; *p* < 0.001). Compared with HP with no pre-season sickness absence, those with 1–3 pre-season sickness absence days had slightly higher adjusted odds of vaccination (aOR 1.08, 95% CI 1.03–1.14), whereas those with ≥8 days had lower adjusted odds (aOR 0.91, 95% CI 0.85–0.97). **Conclusions**: In-season influenza vaccination uptake among HP in Kuwait was low across three consecutive influenza seasons, with limited continuity of annual vaccination. Previous-year vaccination was the strongest predictor of current-season vaccination uptake, while the association between pre-season sickness absence and vaccination varied by absence duration. These findings support continued efforts to improve and sustain in-season influenza vaccination uptake among HP, while further research is needed to clarify the factors underlying vaccination decisions.

## 1. Introduction

Healthcare personnel (HP) are a critical component of resilient health systems, and maintaining a healthy workforce is essential for ensuring continuity of healthcare delivery [1,2]. Seasonal influenza infection remains an important occupational health risk among HP because of their frequent exposure to infected patients and healthcare environments [3,4,5]. Influenza infection among HP can contribute to sickness absence, workforce shortages, and disruption of healthcare services, while also increasing the risk of transmission to vulnerable patients [3]. Globally, seasonal influenza causes approximately 3–5 million cases of severe illness and 290,000–650,000 respiratory deaths annually [6]. Consequently, annual influenza vaccination is recommended for HP by the World Health Organization (WHO) and numerous national public health authorities as a key strategy to protect both HP and patients [3,5,7,8,9]. Furthermore, when vaccine strains are well matched to circulating viruses, influenza vaccination has been shown to be highly effective in preventing influenza infection and reducing its associated workforce impacts, including sickness absence [10]. Despite these recommendations, healthcare-associated influenza transmission remains a significant concern, with studies suggesting that a substantial proportion of influenza cases acquired in healthcare settings may be preventable through effective infection prevention measures, including vaccination [11,12].

HP are prioritized for influenza vaccination to protect themselves from infection and to reduce transmission to colleagues and vulnerable patients at increased risk of severe influenza-related complications [13]. While WHO has endorsed a 75% influenza vaccination coverage target for older adults and people with chronic conditions [14], influenza vaccination uptake among HP remains highly variable across regions and countries. A recent systematic review and meta-analysis of 92 studies from 26 countries reported a pooled vaccination rate of 51.3% in the Middle East, compared with 67.1% in the Americas and 42.5% in Europe [15]. During the 2025–2026 season, vaccination coverage was approximately 45% among National Health Service staff in the United Kingdom [16], while coverage in France was reported at around 34.8% in 2019 [17]. In contrast, substantially higher coverage has been reported in the United States (76.3% in 2024–2025) and Finland (83.7–89.6% across the 2017–2018 to 2019–2020 seasons) [18,19]. Despite longstanding recommendations for annual influenza vaccination, many healthcare systems continue to report coverage levels below recommended targets.

Kuwait operates a centralized, publicly funded healthcare system through the Ministry of Health (MOH), providing a unique opportunity to evaluate influenza vaccination uptake among HP using comprehensive nationwide administrative records. Similar to other Gulf Cooperation Council (GCC) countries, influenza vaccination is provided free of charge to HP in Kuwait. Influenza vaccination is offered annually on a voluntary basis through the MOH Winter Vaccination Campaign to HP and other priority groups at increased risk of influenza-related complications [20]. Nevertheless, vaccination uptake varies considerably across the region. In 2024, reported influenza vaccination coverage among HP was 19.1% in Kuwait, 22% in Bahrain, 31% in Oman, 61% in Qatar, and 79% in Saudi Arabia [21]. Despite the availability of free vaccination programs, published evidence on influenza vaccination uptake among HP in Kuwait remains limited [21]. Furthermore, little is known about patterns of repeated vaccination or the demographic and occupational factors associated with influenza vaccination uptake among HP in Kuwait.

Influenza-related sickness absence represents a major workforce challenge in healthcare settings because staffing shortages may compromise the continuity and quality of patient care. Although HP with influenza-like illness are advised not to report to work, studies indicate that many continue working while symptomatic, potentially increasing opportunities for transmission within healthcare facilities [22,23]. Seasonal influenza vaccination has been associated with reduced influenza-related illness and lower sickness absence among HP, thereby helping maintain workforce capacity during influenza seasons [3,7,24]. However, little is known about whether pre-season sickness absence duration influences subsequent influenza vaccination uptake. Pre-season sickness absence may reflect underlying health status, healthcare-seeking behavior, or workplace engagement, potentially affecting an individual’s likelihood of vaccination. Evidence regarding whether pre-season sickness absence is associated with subsequent influenza vaccination uptake among HP remains limited. Understanding this relationship could inform targeted strategies to improve influenza vaccination coverage. Because sickness absence is routinely recorded in administrative databases, examining its relationship with vaccination uptake may help identify HP who could benefit from targeted vaccination outreach. The availability of longitudinal administrative records across multiple influenza seasons provides a unique opportunity to examine this association.

Despite the importance of influenza prevention, published evidence on influenza vaccination uptake among HP in Kuwait remains limited. Therefore, this nationwide multi-season study aimed to examine patterns of in-season influenza vaccination uptake and associated factors among HP in Kuwait across three consecutive influenza seasons. In addition, the study assessed patterns of repeated vaccination over time and explored the association between pre-season sickness absence duration and subsequent in-season influenza vaccination uptake.

## 2. Materials and Methods

### 2.1. Study Design

This longitudinal observational study used administrative records from the MOH in Kuwait to examine influenza vaccination uptake among HP across three consecutive influenza seasons: 2022/2023, 2023/2024, and 2024/2025.

### 2.2. Data Sources and Study Population

Administrative datasets were obtained from the Kuwait MOH. The datasets included HP demographic characteristics, occupational information, sickness absence records, and influenza vaccination records. Demographic variables included age, sex, nationality, and governorate of residence. Occupational variables included profession and managerial position. Sickness absence records included the date and duration of each sick leave episode, whereas vaccination records included the date of influenza vaccination. The influenza vaccination database contained records from seasons preceding the study period, enabling ascertainment of previous-year vaccination status for all study seasons.

**Figure 1:**
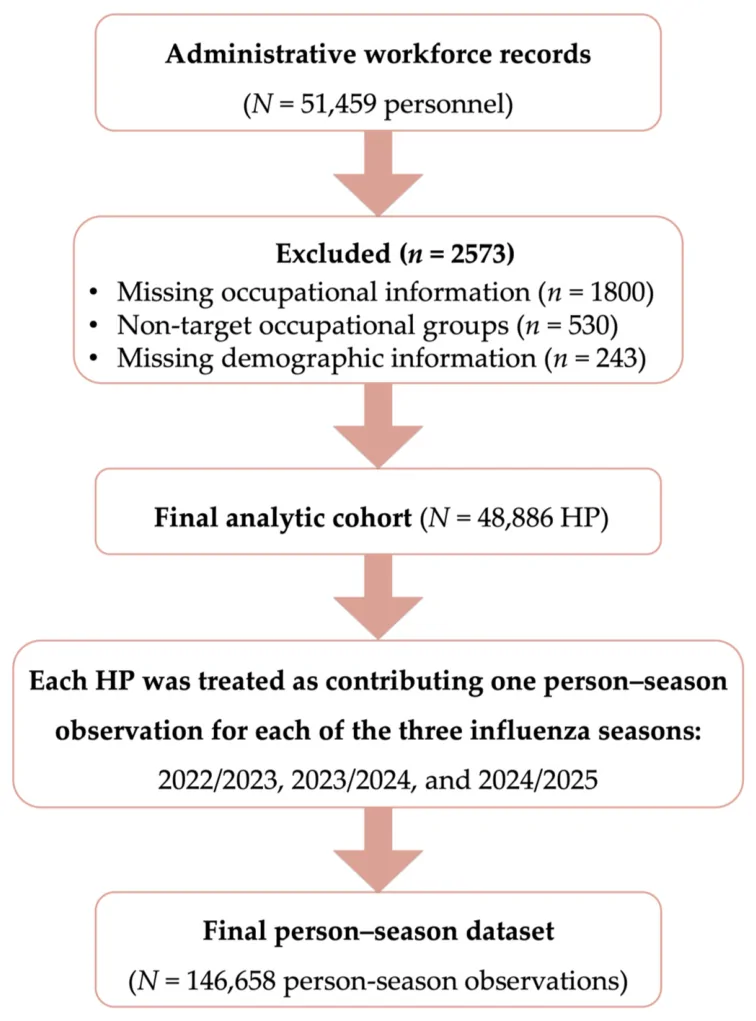
Flow diagram of healthcare personnel cohort selection and construction of the person–season analytic dataset.

For this study, HP comprised physicians, nurses, dentists, pharmacists, and medical technicians employed by the MOH. The initial dataset included 51,459 personnel during the study period. Records with no occupational information (*n* = 1800), records from non-target occupational groups (*n* = 530), including vocational and service workers, assistant technical jobs, administrators, engineering affairs staff, and supporting jobs, and records with missing demographic information (*n* = 243) were excluded. After these exclusions (2573 HP; 5.0% of the initial dataset), the final analytic cohort comprised 48,886 HP, who were treated as contributing 146,658 person–season observations across the three influenza seasons. The final analytic dataset contained complete data for all variables included in the analyses. Therefore, all statistical analyses were performed using a complete-case approach. Figure 1 illustrates the HP cohort selection process and construction of the final person–season dataset.

### 2.3. Unit of Analysis and Multi-Season Person–Season Dataset Construction

The unit of analysis was the person–season. A person–season dataset was constructed, with each HP treated as contributing one observation for each of the three influenza seasons (2022/2023, 2023/2024, and 2024/2025), yielding 146,658 person–season observations. Because annual employment status and workforce entry and exit dates were unavailable, all HP were assumed to be eligible for observation in each season. This structure allowed vaccination uptake to be examined across seasons while accounting for repeated observations within individuals.

### 2.4. Primary Outcome

The primary outcome was in-season influenza vaccination uptake, defined as receipt of an influenza vaccine between September and March of the corresponding influenza season and modeled as a binary outcome (yes/no). This period corresponds to the MOH’s annual Winter Vaccination Campaign and the months of increased seasonal respiratory virus activity in Kuwait [20,25]. Vaccination status was determined from administrative vaccination records, with HP classified as vaccinated if they had at least one vaccination record during this period. Vaccinations recorded outside this period (April–August) were classified as not vaccinated for the primary outcome analysis.

### 2.5. Independent Variables

Demographic variables included age, sex (male or female), nationality (Kuwaiti or non-Kuwaiti), and governorate of residence (Ahmadi, Asimah, Farwaniya, Hawalli, Jahra, and Mubarak Al-Kabeer). Age was calculated from the date of birth at the beginning of each influenza season (1 September) and was analyzed as a continuous variable.

Profession was categorized as physician, nurse, dentist, pharmacist, or medical technician, in line with the MOH workforce classification system [26]. In the MOH administrative records, the medical technician category encompassed technicians working across a range of allied health and technical specialties, including medical laboratory sciences, radiology, nutrition and feeding, physiotherapy, pharmacy support services, emergency medical services, environmental health, anesthesia, sterilization, dental technical services, speech and hearing therapy, occupational therapy, respiratory therapy, psychotherapy, and other allied health technical specialties.

Managerial position was categorized as managerial versus non-managerial. Managerial position and governorate of residence were treated as time-invariant variables because updated time-varying records were not available in the administrative datasets. Consequently, changes in managerial status or residence during the study period could not be captured.

Previous-year influenza vaccination status was defined using MOH vaccination records from the preceding September–August period and analyzed as a binary variable. HP with at least one influenza vaccination record during this period were classified as previously vaccinated, whereas those without a record were classified as not previously vaccinated. For the 2022/2023 study season, previous-year vaccination status was determined using records from September 2021 through August 2022. The corresponding periods were September 2022 through August 2023 for the 2023/2024 season and September 2023 through August 2024 for the 2024/2025 season.

#### Measurement of Pre-Season Sickness Absence

Pre-season sickness absence duration was defined as the cumulative number of sick leave days recorded in administrative records during the period from January through August preceding each influenza season. This time window was selected to establish temporal precedence between pre-season sickness absence and subsequent in-season influenza vaccination uptake. Sickness absence records included physician-certified sick leave issued by MOH facilities and electronic sick leave obtained without a medical consultation through Sahel, Kuwait’s unified government application, which enables eligible employees to request and obtain short-term sick leave electronically [27]. Pre-season sickness absence duration was categorized as 0, 1–3, 4–7, and ≥8 days. These categories were selected to examine potential non-linear associations between pre-season sickness absence and subsequent influenza vaccination uptake.

### 2.6. Statistical Analysis

Descriptive statistics were used to summarize HP characteristics according to in-season influenza vaccination status. Age was summarized using means and standard deviations (SDs), while categorical variables were summarized using frequencies and percentages. Season-specific in-season influenza vaccination uptake was calculated for each influenza season as the proportion of HP vaccinated during the campaign period (September–March). Furthermore, to evaluate longitudinal vaccination consistency, HP were categorized according to the number of influenza seasons in which they received an in-season influenza vaccine: never vaccinated, vaccinated in one season, vaccinated in two seasons, or consistently vaccinated across all three seasons.

Generalized estimating equations (GEE) logistic regression with a logit link, binomial distribution, exchangeable correlation structure, and robust standard errors clustered by HP was used to identify factors associated with in-season influenza vaccination uptake while accounting for repeated observations across influenza seasons. Univariable GEE models were first fitted to estimate crude odds ratios (cORs) and corresponding 95% confidence intervals (CIs) for each independent variable. A multivariable GEE model including all covariates was then fitted to estimate adjusted odds ratios (aORs) and corresponding 95% CIs. Independent variables included age, sex, nationality, governorate of residence, profession, managerial position, previous-year vaccination status, pre-season sickness absence duration, and influenza season. Multicollinearity among independent variables was assessed using variance inflation factors (VIFs). Sensitivity analyses were performed using mixed-effects logistic regression models with random intercepts for HP.

To further evaluate the relationship between pre-season sickness absence duration and vaccination uptake, adjusted predicted probabilities with corresponding 95% CIs were estimated from the multivariable GEE model and graphically presented. Statistical significance was defined as a two-sided *p*-value < 0.05. Data management and statistical analyses were conducted using Stata version 14.2 (StataCorp LLC, College Station, TX, USA).

## 3. Results

Of the 51,459 HP initially identified, 2573 (5.0%) were excluded due to missing occupational information, inclusion in non-target occupational categories, or missing demographic information, resulting in a final analytic cohort comprising 48,886 HP, each treated as contributing one observation to each of the three influenza seasons, yielding 146,658 person–season observations. Table 1 presents the characteristics of HP by in-season influenza vaccination status across the pooled study seasons. Overall, in-season influenza vaccination was recorded in 12,787 of the 146,658 person–season observations (8.7%). The mean age across all person–season observations was 37.8 years (SD = 10.1), compared with 41.8 years (SD = 10.7) among vaccinated person–season observations. Male HP demonstrated higher vaccination prevalence than females (10.4% vs. 7.7%), while non-Kuwaiti personnel had higher uptake compared with Kuwaiti personnel (9.8% vs. 6.1%). Vaccination uptake differed by profession, with physicians demonstrating the highest uptake (12.6%), followed by nurses (9.4%). Uptake was substantially lower among dentists (5.8%), pharmacists (5.2%), and medical technicians (5.1%). Geographical variation was also observed, with Hawalli governorate of residence showing the highest vaccination uptake (10.6%) and Jahra the lowest (5.9%). In addition, managerial personnel demonstrated higher vaccination uptake than non-managerial personnel (11.92% vs. 8.68%). In-season influenza vaccination uptake was highest and nearly identical among HP with no pre-season sickness absence and those with 1–3 days (9.8% in each group). Uptake decreased with longer pre-season sickness absence duration, from 7.7% among those with 4–7 days to 5.7% among those with ≥8 days. In-season influenza vaccination uptake also differed by previous vaccination status. HP who had received an influenza vaccine in the previous year demonstrated substantially higher uptake during the current season than those who had not (38.5% vs. 6.7%).

Table 2 presents the season-specific in-season influenza vaccination uptake among HP across the three influenza seasons. Vaccination uptake demonstrated a gradual increasing trend over time, rising from 7.23% in the 2022/2023 season to 8.99% in the 2023/2024 season and 9.94% in the 2024/2025 season. Despite this modest increase, overall, in-season influenza vaccination uptake remained low throughout the study period.

Table 3 presents repeated in-season influenza vaccination patterns among HP across the three influenza seasons. Overall, the majority of HP (81.7%) did not receive an in-season influenza vaccine during any of the study seasons. Vaccination in only one season was observed in 12.2% of HP, whereas 4.3% received vaccination in two seasons. Consistent in-season vaccination across all three influenza seasons was uncommon, occurring in only 1.8% of HP. Collectively, these findings demonstrate low continuity of annual influenza vaccination uptake among HP over the study period.

Table 4 presents the crude and multivariable GEE logistic regression analyses examining factors associated with in-season influenza vaccination uptake among HP. No evidence of problematic multicollinearity was identified among the independent variables (mean VIF: 1.62; all VIFs < 3.1). Several associations observed in crude analyses attenuated after multivariable adjustment, suggesting confounding by demographic and occupational characteristics. Increasing age remained independently associated with higher adjusted odds of vaccination uptake (aOR 1.029, 95% CI 1.027–1.032; *p* < 0.001). Female HP had lower adjusted odds of receiving in-season influenza vaccination than male HP (aOR 0.87, 95% CI 0.82–0.91; *p* < 0.001), whereas non-Kuwaiti HP had higher adjusted odds than Kuwaiti HP (aOR 1.31, 95% CI 1.22–1.40; *p* < 0.001). Significant regional variation in vaccination uptake was observed across governorates, although some regional associations attenuated following adjustment. Compared with physicians, nurses, dentists, pharmacists, and medical technicians had significantly lower adjusted odds of vaccination uptake (all *p* < 0.001). Previous-year influenza vaccination was the strongest independent predictor of current-season vaccination uptake (aOR 2.01, 95% CI 1.87–2.16; *p* < 0.001).

Pre-season sickness absence duration was also associated with vaccination uptake. Compared with HP with no pre-season sickness absence, those with 1–3 days had slightly higher adjusted odds of vaccination uptake (aOR 1.08, 95% CI 1.03–1.14; *p* = 0.003), whereas those with ≥8 days had lower adjusted odds (aOR 0.91, 95% CI 0.85–0.97; *p* = 0.004). No statistically significant association was observed among HP with 4–7 days of pre-season sickness absence (aOR 0.99, 95% CI 0.93–1.05; *p* = 0.719). Although managerial personnel showed higher vaccination uptake in crude analyses, this association was no longer statistically significant after adjustment. Compared with the 2022/2023 influenza season, the adjusted odds of in-season vaccination uptake were significantly higher during the 2023/2024 and 2024/2025 seasons. Sensitivity analyses using mixed-effects logistic regression yielded findings consistent with the primary GEE analysis (Supplementary Table S1).

**Figure 2:**
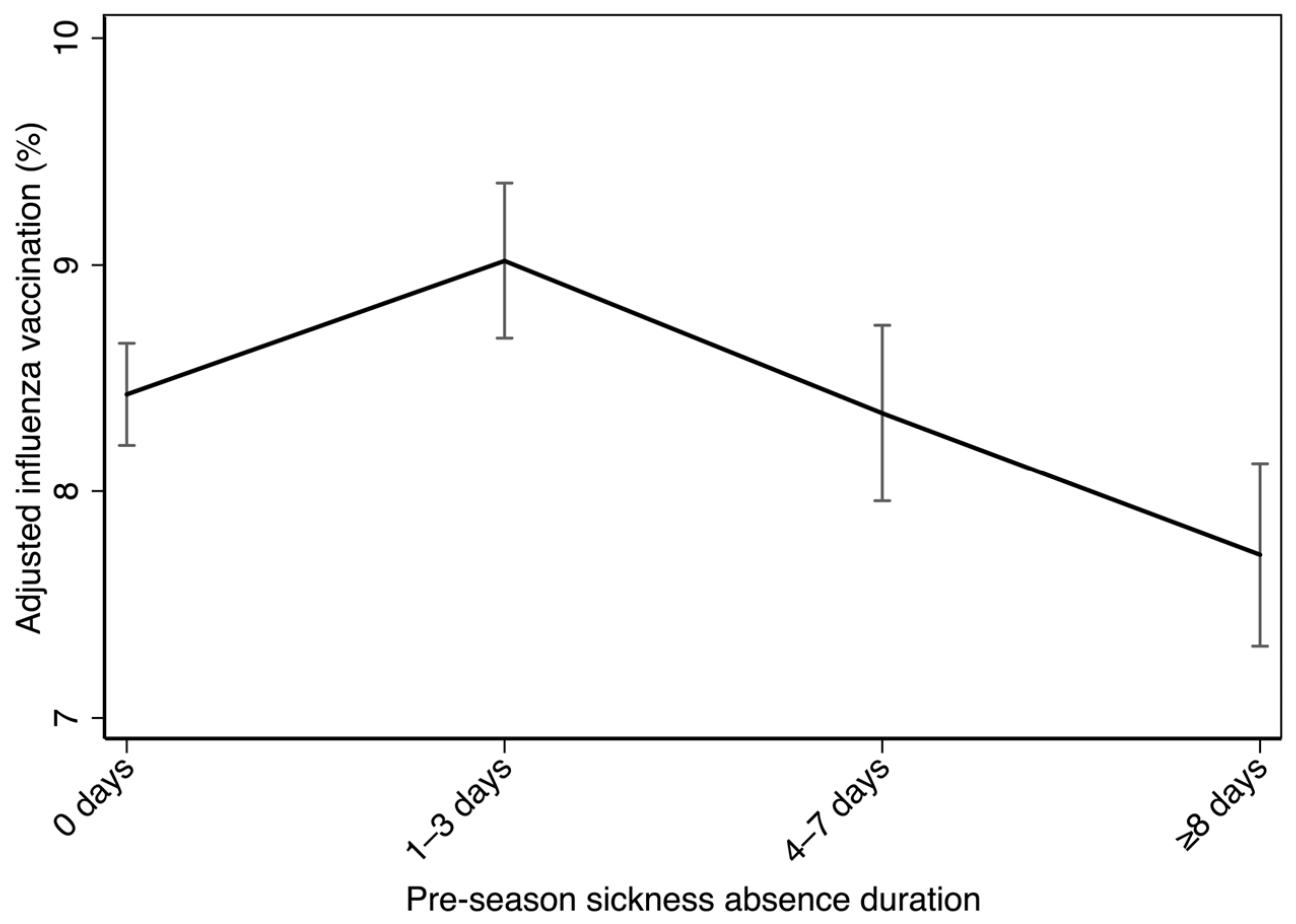
Adjusted probability of in-season influenza vaccination according to pre-season sickness absence duration (January–August preceding each influenza season) among healthcare personnel in Kuwait.

Figure 2 illustrates the adjusted predicted probability of in-season influenza vaccination according to pre-season sickness absence duration derived from the multivariable GEE model. Although vaccination uptake was similar between HP with no pre-season sickness absence and those with 1–3 days of pre-season sickness absence (Table 1), the adjusted predicted probability was slightly in the latter group after adjustment for the other variables included in the model. Vaccination uptake demonstrated a non-linear pattern, with the highest adjusted probability observed among HP with 1–3 days of pre-season sickness absence. In contrast, lower adjusted vaccination probabilities were observed among HP with longer durations of pre-season sickness absence, particularly among those with ≥8 days. These findings were consistent with the multivariable GEE regression analysis.

## 4. Discussion

Despite ongoing national seasonal influenza vaccination campaigns, in-season influenza vaccination uptake among HP in Kuwait remained low across three consecutive influenza seasons. Previous-year vaccination emerged as the strongest predictor of current-season vaccination uptake, highlighting the importance of habitual vaccination behavior. In addition, vaccination uptake varied according to demographic and occupational characteristics, while pre-season sickness absence duration demonstrated a non-linear association with vaccination uptake, with lower uptake observed among HP with longer durations of pre-season sickness absence. Although several associations reached statistical significance, some were modest in magnitude and should therefore be interpreted cautiously with respect to their practical importance.

Compared with the previously reported influenza vaccination coverage estimate of 19.1% among HP in Kuwait [21], uptake was lower in the present study. However, differences in the periods used to ascertain vaccination and in the definitions of the study populations limit direct comparability. Nevertheless, both the previous estimate and the findings of the present study indicate that influenza vaccination coverage among HP in Kuwait remains lower than that reported in several other countries [17,18,19,21,28]. These findings indicate considerable scope for improving influenza vaccination coverage among the healthcare workforce in Kuwait.

Seasonal influenza vaccination is widely available and provided free of charge through Kuwait’s public healthcare system, and HP are recognized as a priority group for vaccination [9,25]. National MOH guidance identifies HP as a target group, directs healthcare facilities to coordinate their vaccination, and provides for electronic registration and periodic reporting of vaccination activity [25]. However, the guidance does not describe influenza vaccination as a condition of employment, specify consequences or incentives related to vaccination status, or establish formal procedures for documenting vaccine declination. Information on the nature and consistency of vaccination-promotion activities across individual healthcare facilities, and whether vaccination data were routinely used for audit and feedback, was unavailable. Therefore, the persistently low vaccination uptake observed in this study may reflect not only individual-level factors, such as vaccine confidence and perceived risk, but also variation in the implementation and intensity of institutional vaccination-promotion strategies.

Regional evidence supports the potential importance of institutional factors. A recent hospital-based study in Abu Dhabi identified inconvenient vaccination times, limited policy awareness, and inadequate administrative follow-up as barriers to influenza vaccination among HP [29]. Multifaceted strategies combining accessible on-site vaccination, active communication and education, formal declination procedures, and peer vaccination have been associated with higher influenza vaccination uptake among HP across diverse healthcare settings, including Qatar [30,31]. Relevant, although indirect, contextual evidence comes from a recent qualitative study of maternal influenza vaccination in Kuwait, in which preventive medicine professionals, including policymakers and vaccination campaign managers, identified vaccination champions as important facilitators and emphasized healthcare professional education and clear national policies and guidelines as strategies for improving vaccination provision and uptake [32].

Repeated in-season influenza vaccination was uncommon among HP in Kuwait. Similarly, a four-year prospective cohort study in Lima, Peru, found limited continuity of annual influenza vaccination among HP [33]. Nevertheless, previous-year influenza vaccination was the strongest positive predictor of current-season uptake. HP with a recorded influenza vaccination in the preceding year had approximately twice the adjusted odds of current-season vaccination compared with those without a previous-year vaccination record. Previous studies have similarly identified past vaccination behavior as an important predictor of subsequent influenza vaccination uptake across studies of HP and the general population [34,35,36]. Related evidence from Jordan identified previous influenza vaccination experience as the strongest predictor of future influenza vaccination motivation among healthcare workers [37]. Previous vaccination may reflect greater confidence in vaccine safety and effectiveness, as well as an established pattern of vaccination behavior, although these factors were not measured in the present study [34,38]. These findings suggest the potential value of strategies that promote both initial and repeated annual vaccination. However, further research is needed to determine whether encouraging first-time vaccination leads to sustained annual uptake in practice.

Influenza vaccination uptake varied substantially across professional groups in this study, with physicians more likely to be vaccinated than nurses, dentists, pharmacists, and medical technicians. Although physicians had the highest uptake, coverage remained considerably lower than that reported regionally, such as among primary care physicians in Qatar [39]. Differences in vaccination uptake across professional groups have been reported among HP in diverse healthcare settings [15,21,36,40,41]. Differences in knowledge, perceived risk, confidence in vaccine effectiveness and safety, social and professional norms, and access to vaccination services may contribute to these variations [42]. These findings may help inform workforce vaccination strategies tailored to specific professional groups rather than relying solely on universal approaches.

Older HP, males, and non-Kuwaitis were more likely to receive influenza vaccination. Similar age-related patterns have been reported previously and may reflect greater awareness of personal health risks and accumulated professional experience [15,36,40,43]. The higher vaccination uptake observed among male HP is also consistent with previous evidence indicating that male HP are generally more likely to receive influenza vaccination than female HP [36,40,41,42,44,45,46], although the reasons underlying this difference remain unclear. Higher vaccination uptake among non-Kuwaiti HP may reflect differences in workforce composition, occupational expectations, or preventive health behaviors; however, the administrative data used in this study did not permit exploration of the mechanisms underlying this association. Similar nationality-related differences have been reported in Saudi Arabia, where lower vaccination uptake was observed among Saudi compared with non-Saudi HP [47]. Further research is needed to understand potential barriers to vaccination among Kuwaiti HP. These findings may help inform targeted vaccination strategies directed toward demographic groups with consistently lower vaccination uptake.

Pre-season sickness absence was also independently associated with subsequent influenza vaccination uptake, although this pattern was not linear. Compared with HP with no pre-season sickness absence, those with 1–3 days had slightly higher adjusted odds of vaccination, whereas those with ≥8 days had lower adjusted odds. Evidence on the association between pre-season sickness absence and subsequent influenza vaccination uptake remains limited, as previous research has primarily examined the reverse temporal relationship, demonstrating lower subsequent sickness absence among vaccinated than unvaccinated HP at the individual level and an inverse association between organizational vaccination coverage and staff sickness absence [41,48]. The present study contributes to this limited evidence by examining the association between pre-season sickness absence duration and subsequent in-season influenza vaccination uptake across three consecutive influenza seasons using administrative data.

Several alternative explanations related to health status and workplace participation should also be considered when interpreting these findings. Although sickness absence was measured before the vaccination season, greater cumulative pre-season sickness absence may have been a marker of underlying or chronic health conditions, healthcare utilization patterns, or occupational circumstances that persisted into the vaccination season and influenced subsequent vaccination opportunities. However, information on these factors, as well as modified work schedules, extended leave, workplace attendance during the vaccination campaign, and the underlying reasons for sickness absence, was unavailable. Their contribution to the observed findings therefore could not be evaluated. Consequently, the observed association should not be interpreted as evidence that pre-season sickness absence itself reduced vaccination uptake.

Although statistically significant associations were observed between pre-season sickness absence duration and subsequent in-season influenza vaccination uptake for some duration categories, their magnitudes were modest and should be interpreted cautiously in terms of practical importance. Pre-season sickness absence should therefore not be considered a stand-alone indicator for targeting vaccination interventions. Its potential value as complementary information alongside demographic, occupational, and health-related factors remains to be established. Further studies should evaluate whether incorporating sickness absence information into occupational health strategies improves influenza vaccination uptake in practice.

The persistently low influenza vaccination uptake observed in this study highlights the need for multifaceted strategies among HP in Kuwait. Vaccination behavior may be influenced by factors such as confidence in vaccine safety and effectiveness, perceived benefits, previous vaccination experiences, and professional norms [38]. Consistent with these factors, a survey of 390 HP in Oman identified self-protection and protection of others as the main motivators for vaccination, while fear of side effects was the leading reason for hesitancy [49]. Systematic reviews suggest that multifaceted workplace vaccination programs combining education, convenient vaccine access, leadership support, organizational policies, and vaccination champions may achieve greater improvements in influenza vaccination uptake among HP than education or promotion alone [50,51]. The strong association between previous-year and current-season vaccination in the present study suggests continuity in vaccination behavior; however, it does not establish that first-time vaccination leads to subsequent annual vaccination. Strategies that promote both initial and repeated vaccination therefore warrant further evaluation. If effective, such strategies could improve vaccination coverage and support workforce resilience and continuity of healthcare services in Kuwait.

This study has several important strengths. The use of nationwide MOH administrative records enabled the inclusion of nearly 49,000 HP and provided a comprehensive assessment of in-season influenza vaccination uptake and associated factors within Kuwait’s public healthcare workforce. Person–level linkage across seasons allowed the assessment of repeated vaccination patterns and the examination of the association between previous-year and subsequent vaccination. Administrative vaccination records reduced the potential for recall and self-report bias. In addition, information on the timing and cumulative duration of sickness absence allowed the examination of the relatively understudied association between pre-season sickness absence and subsequent vaccination uptake. Finally, the broadly consistent findings from the mixed-effects sensitivity analysis supported the robustness of the primary GEE results.

Despite these strengths, the study has several limitations. First, the administrative databases did not capture several behavioral and clinical factors potentially associated with vaccination uptake, including vaccine hesitancy, vaccination attitudes, perceived risk, knowledge, and chronic medical conditions. Information on work schedules, extended leave, workplace attendance, and healthcare utilization was also unavailable. These unmeasured factors may have influenced vaccination uptake and limited interpretation of the observed associations. Second, diagnostic information was unavailable for electronic self-certified sick leave. Pre-season sickness absence was therefore analyzed as cumulative all-cause duration, preventing examination of diagnosis-specific associations with vaccination uptake. Third, because annual employment status and workforce entry and exit dates were unavailable, all HP were treated as eligible for observation in each influenza season. This assumption may have resulted in misclassification of seasonal workforce eligibility and affected the estimated vaccination patterns and associations, although the extent of any effect is unknown. Fourth, managerial position and governorate of residence were treated as time-invariant because changes in these characteristics during the study period were not captured. Finally, although the Kuwait MOH employs the majority of HP in the country [26], the findings may not be fully generalizable to personnel working in other healthcare sectors, particularly the private sector.

## 5. Conclusions

In-season influenza vaccination uptake among HP in Kuwait was low across three consecutive influenza seasons, with limited continuity of vaccination across multiple seasons. In-season influenza vaccination uptake was associated with several demographic and occupational characteristics, previous vaccination, and pre-season sickness absence duration, although some associations were modest in magnitude. Improving influenza vaccination uptake among HP should be considered a workforce health priority. Targeted, multifaceted occupational health interventions that promote initial uptake and sustained annual vaccination may help protect workforce health and support the continuity of healthcare services during seasonal influenza epidemics. Further research is needed to better understand the individual and institutional factors underlying vaccination decisions and the continuity of annual vaccination among HP in Kuwait.

## Figures and Tables

**Table 1:** Characteristics of healthcare personnel and in-season influenza vaccination uptake across three consecutive influenza seasons in Kuwait (pooled person–season observations).

Variable	Total Person–Season *n* (%)	Vaccinated Person–Season *n* (%) ^‡^
Age (years), mean (SD)	37.8 (10.1)	41.8 (10.7) ^†^
Sex	—	—
Male	54,492 (37.16)	5658 (10.38)
Female	92,166 (62.84)	7129 (7.73)
Nationality	—	—
Kuwaiti	43,464 (29.64)	2645 (6.09)
Non-Kuwaiti	103,194 (70.36)	10,142 (9.83)
Governorate of residence	—	—
Asimah	17,067 (11.64)	1405 (8.23)
Ahmadi	20,001 (13.64)	1579 (7.89)
Farwaniya	44,550 (30.38)	4025 (9.03)
Hawalli	40,497 (27.61)	4306 (10.63)
Jahra	18,108 (12.35)	1074 (5.93)
Mubarak Al-Kabeer	6435 (4.39)	398 (6.18)
Profession	—	—
Physician	34,530 (23.54)	4343 (12.58)
Nurse	61,500 (41.93)	5807 (9.44)
Dentist	8211 (5.60)	472 (5.75)
Pharmacist	6993 (4.77)	365 (5.22)
Medical technician	35,424 (24.15)	1800 (5.08)
Managerial position	—	—
No	144,846 (98.76)	12,571 (8.68)
Yes	1812 (1.24)	216 (11.92)
Previous-year influenza vaccination	—	—
No	137,509 (93.76)	9264 (6.74)
Yes	9149 (6.24)	3523 (38.51)
Pre-season sickness absence duration (days)	—	—
0	69,278 (47.24)	6775 (9.78)
1–3	27,752 (18.92)	2716 (9.79)
4–7	23,066 (15.73)	1782 (7.73)
≥8	26,562 (18.11)	1514 (5.70)

SD: standard deviation. ^‡^ Percentage vaccinated within each category (row percentage). ^†^ Mean age among vaccinated person–season observations. Notes: The unit of analysis was person–season (*N* = 146,658 person–season observations). Overall, in-season influenza vaccination uptake was 8.7% (12,787/146,658 person–season observations). In-season vaccination was defined as receipt of an influenza vaccine from September through March. Previous-year influenza vaccination was defined as receipt of an influenza vaccine during the preceding September–August period.

**Table 2:** In-season influenza vaccination uptake among healthcare personnel across three influenza seasons in Kuwait (*N* = 48,886 HP per season).

Season	Uptake (%)
2022/2023	7.23
2023/2024	8.99
2024/2025	9.94

Notes: In-season influenza vaccination was defined as receipt of an influenza vaccine from September through March. Vaccinations recorded from April through August were not counted as in-season vaccinations.

**Table 3:** Repeated in-season influenza vaccination patterns among healthcare personnel across three influenza seasons (*N* = 48,886).

Vaccination Pattern	*n*	%
Never vaccinated	39,960	81.7
Vaccinated in one season	5952	12.2
Vaccinated in two seasons	2087	4.3
Vaccinated in all three seasons	887	1.8

Notes: In-season influenza vaccination was defined as receipt of an influenza vaccine from September through March of each influenza season (2022/2023, 2023/2024, and 2024/2025). Vaccination patterns were classified according to the number of study seasons in which each healthcare personnel member received an in-season influenza vaccine.

**Table 4:** Crude and adjusted odds ratios for factors associated with in-season influenza vaccination uptake among healthcare personnel, estimated using GEE (146,658 person–season observations).

Variable		cOR (95% CI)	*p*-Value	aOR (95% CI)	*p*-Value
Age (Years)		1.042 (1.039–1.044)	<0.001 *	1.029 (1.027–1.032)	<0.001 *
Sex	Male	Reference	—	Reference	—
	Female	0.72 (0.69–0.76)	<0.001 *	0.87 (0.82–0.91)	<0.001 *
Nationality	Kuwaiti	Reference		Reference	
	Non-Kuwaiti	1.68 (1.59–1.78)	<0.001 *	1.31 (1.22–1.40)	<0.001 *
Governorate of residence	Asimah	Reference	—	Reference	—
	Ahmadi	0.96 (0.87–1.05)	0.358	0.79 (0.72–0.87)	<0.001 *
	Farwaniya	1.11 (1.02–1.20)	0.016 *	0.86 (0.78–0.93)	<0.001 *
	Hawalli	1.33 (1.22–1.44)	<0.001 *	1.00 (0.92–1.09)	0.967
	Jahra	0.70 (0.63–0.78)	<0.001 *	0.70 (0.63–0.78)	<0.001 *
	Mubarak Al-Kabeer	0.73 (0.63–0.85)	<0.001 *	0.87 (0.75–1.00)	0.056
Profession	Physician	Reference	—	Reference	—
	Nurse	0.72 (0.69–0.76)	<0.001 *	0.78 (0.74–0.83)	<0.001 *
	Dentist	0.42 (0.37–0.48)	<0.001 *	0.56 (0.49–0.63)	<0.001 *
	Pharmacist	0.38 (0.33–0.44)	<0.001 *	0.49 (0.42–0.56)	<0.001 *
	Medical technician	0.37 (0.35–0.40)	<0.001 *	0.49 (0.46–0.53)	<0.001 *
Managerial position	No	Reference	—	Reference	—
	Yes	1.42 (1.18–1.73)	<0.001 *	1.06 (0.88–1.28)	0.544
Previous-year influenza vaccination	No	Reference		Reference	
	Yes	2.24 (2.07–2.42)	<0.001 *	2.01 (1.87–2.16)	<0.001 *
Pre-season sickness absence duration (days)	0	Reference	—	Reference	—
	1–3	1.04 (0.99–1.09)	0.13	1.08 (1.03–1.14)	0.003 *
	4–7	0.86 (0.81–0.91)	<0.001 *	0.99 (0.93–1.05)	0.719
	≥8	0.70 (0.66–0.74)	<0.001 *	0.91 (0.85–0.97)	0.004 *
Influenza season	2022/2023	Reference	—	Reference	—
	2023/2024	1.27 (1.22–1.32)	<0.001 *	1.09 (1.04–1.14)	<0.001 *
	2024/2025	1.42 (1.36–1.47)	<0.001 *	1.16 (1.11–1.21)	<0.001 *

GEE: generalized estimating equations; cOR: crude odds ratio; aOR: adjusted odds ratio; CI: confidence interval. * indicates statistical significance at *p* < 0.05. Notes: Generalized estimating equations with a logit link, binomial distribution, exchangeable correlation structure, and robust standard errors were used. The outcome was in-season influenza vaccination uptake, defined as receipt of influenza vaccination during September–March. The unit of analysis was person–season (146,658 person–season observations from 48,886 healthcare personnel). Reference categories were male, Kuwaiti, Asimah Governorate, physician, non-managerial position, no previous-year influenza vaccination (defined as no influenza vaccination during the preceding influenza year [September–August]), no pre-season sickness absence (0 days), and the 2022/2023 influenza season.

## Data Availability

The data presented in this study are available upon reasonable request from the corresponding author. The data are not publicly available due to legal and administrative restrictions imposed by the Kuwait Ministry of Health. Access to the data is subject to approval by the Kuwait Ministry of Health.

## Supplementary Materials

The following supporting information can be downloaded at https://www.mdpi.com/article/10.3390/vaccines14080712/s1, Table S1: Sensitivity analysis of factors associated with in-season influenza vaccination uptake using mixed-effects logistic regression.

## Abbreviations

The following abbreviations are used in this manuscript:

HP	Healthcare personnel
MOH	Ministry of Health
SD	Standard deviation
cORs	Crude odds ratios
aORs	Adjusted odds ratios
GEE	Generalized estimating equation
CIs	Confidence intervals
VIFs	Variance inflation factors
