# Supplementary material for: Patterns and Predictors of Influenza Vaccination Uptake Among Healthcare Personnel in Kuwait: A Nationwide Multi-Season Study
Source: Vaccines (Basel). 2026 Aug 19;14(8):712. doi: 10.3390/vaccines14080712 (PMC13517409; doi:10.3390/vaccines14080712)
Supplement: Supplementary file 1 [file vaccines-14-00712-s001.zip › vaccines-4471769-supplementary.pdf]

Supplemental Table S1. Sensitivity analysis of factors associated with in-season influenza vaccination uptake using mixed-effects logistic regression.

Variable		aOR	95% CI	P-value
Age (years)		1.039	1.035–1.043	<0.001 *
Sex	Male	Reference	—	—
	Female	0.82	0.76–0.87	<0.001 *
Nationality	Kuwaiti	Reference	—	—
	Non-Kuwaiti	1.40	1.27–1.53	<0.001 *
Governorate of residence	Asimah	Reference	—	—
	Ahmadi	0.75	0.66–0.86	<0.001 *
	Farwaniya	0.82	0.73–0.93	<0.001 *
	Hawalli	1.05	0.94–1.18	0.352
	Jahra	0.63	0.54–0.72	<0.001 *
	Mubarak Al-Kabeer	0.87	0.72–1.05	0.124
Profession	Physician	Reference	—	—
	Nurse	0.74	0.68–0.80	<0.001 *
	Dentist	0.43	0.37–0.51	<0.001 *
	Pharmacist	0.39	0.32–0.46	<0.001 *
	Medical technician	0.39	0.34–0.42	<0.001 *
Managerial position	No	Reference	—	—
	Yes	1.08	0.83–1.40	0.547
Previous-year influenza vaccination	No	Reference	—	—
	Yes	1.63	1.42–1.86	<0.001 *
Pre-season sickness absence duration (days)	0	Reference	—	—
	1–3	1.12	1.05–1.20	0.001 *
	4–7	0.99	0.92–1.08	0.874
	≥8	0.89	0.82–0.97	0.009 *
Influenza season	2022/2023	Reference	—	—
	202/2024	1.20	1.13–1.28	<0.001 *
	2024/2025	1.32	1.23–1.42	<0.001 *

aOR: adjusted odds ratio; CI: confidence interval; *: statistically significant at $p < 0.05$. Notes: Mixed-effects logistic regression with a random intercept for healthcare personnel ID to account for repeated observations. The outcome was in-season influenza vaccination uptake defined as receipt of influenza vaccination during September–March. The unit of analysis was the person–season (146,658 person–season observations from 48,886 healthcare personnel). Reference categories were male, Kuwaiti, Asimah Governorate, physician, non-managerial position, no previous-year influenza vaccination (defined as no influenza vaccination during the preceding influenza year [September–August]), no pre-season sickness absence (0 days), and the 2022/2023 influenza season.
